# CeSpGRN: inferring cell-specific gene regulatory networks from single-cell multi-omics and spatial data

**DOI:** 10.1093/bioinformatics/btag324

**Published:** 2026-06-01

**Authors:** Ziqi Zhang, Jongseok Han, Le Song, Xiuwei Zhang

**Affiliations:** School of Computational Science and Engineering, Georgia Institute of Technology, Atlanta, GA 30332, United States; School of Computational Science and Engineering, Georgia Institute of Technology, Atlanta, GA 30332, United States; Mohamed bin Zayed University of Artificial Intelligence, Abu Dhabi, United Arab Emirates; GenBio AI, Palo Alto, CA, 94301, United States; School of Computational Science and Engineering, Georgia Institute of Technology, Atlanta, GA 30332, United States

## Abstract

**Motivation:**

Single-cell sequencing technologies allow researchers to study cell-cell variation within a cell population. Variations between cells are driven by the underlying biological network, particularly gene regulatory networks (GRNs). GRNs rewire as cells evolve, and different cells can have different GRNs. However, while single-cell RNA-sequencing (scRNA-seq) and single-cell multi-omics data have been used to reconstruct GRNs, the output GRNs are rarely cell-specific, but rather, most existing methods infer population-level or cell-type-level GRNs.

**Results:**

We propose CeSpGRN (Cell-Specific Gene Regulatory Network inference), a method that infers cell-specific GRNs from scRNA-seq, paired scRNA-seq and scATAC-seq, or spatial transcriptomic data. In particular, existing methods that use matching scRNA-seq and scATAC-seq data incorporate population-level region information in GRN inference, whereas CeSpGRN utilizes single-cell resolution region information. CeSpGRN infers cell-specific GRNs using a kernel-weighted Gaussian Copula Graphical Model, and incorporates multi-omic or spatial location information when constructing the objective function. We tested CeSpGRN on both simulated and real datasets, and the results show that CeSpGRN has a superior performance compared to baseline methods in reconstructing GRNs and detecting regulatory interactions that differ between cells. CeSpGRN uncovered regulatory interactions that rewire during biological processes on real datasets.

**Availability and implementation:**

CeSpGRN is a Python package available at https://github.com/PeterZZQ/CeSpGRN.

## 1 Introduction

Gene regulatory networks (GRNs) represent how genes regulate each other during biological processes. Inferring GRNs from gene expression data has been a long-standing and challenging problem. Single-cell gene expression data have been used to infer GRNs, where each cell is used as a sample (Chan *et al.* 2017, Matsumoto *et al.* 2017, Aibar *et al.* 2017, Chen and Mar 2018, Papili Gao *et al.* 2018, Pratapa *et al.* 2020, Zhang *et al.* 2023). These methods aim to learn one GRN from the gene expression data of a population of single cells from either a cell cluster or all the cells in one dataset. However, it has been reported that GRNs are highly dynamic and their topologies change over a temporal process or a spatial landscape (Luscombe *et al.* 2004, Ahmed and Xing 2009, Song *et al.* 2009, Przytycka *et al.* 2010, Kouno *et al.* 2013, Kim *et al.* 2020). Each cell can have its unique GRN according to its developmental stage along the dynamic process. Obtaining cell-specific GRNs is of great significance as it allows researchers to investigate the changes in GRNs during a dynamic process or across a spatial landscape. Dictys (Wang *et al.* 2023a) was proposed to infer time-varying GRNs along the differentiation trajectory using single-cell gene expression data. The method moves a window along the trajectory and infers the GRN for each window step. However, the method does not infer GRNs at single-cell resolution, and requires that cells are pre-ordered by pseudotime, but the pseudotime inference step can also introduce errors. Dai *et al.* developed CSN, which attempts to calculate pairwise gene-gene association in single cells using a statistical measurement (Dai *et al.* 2019). However, CSN analyzes the association for each gene pair independently. In GRN inference, all genes are considered at the same time, which is more challenging than pairwise gene-gene association analysis. Based on CSN, c-CSN (Li *et al.* 2021) is then proposed, which considers the effect of other genes and eliminates the indirect interactions between genes. p-CSN takes one step further and reduces the high false-negative rate of c-CSN using partial independence of statistics (Wang *et al.* 2023b). LocCSN (Wang *et al.* 2021), on the other hand, proposes a localized population construction method for correlation-test, which reduces the false positive edges detected by the CSN algorithm.

Meanwhile, the advance of single-cell multi-omics sequencing technology makes it possible to infer a more accurate GRN using multi-modality information. Recently, methods have been proposed that infer GRNs using jointly profiled scATAC-seq and scRNA-seq data. SCENIC+ (Bravo González-Blas *et al.* 2023) expands upon SCENIC (Aibar *et al.* 2017) and integrates the TF-target information encoded in scATAC-seq data into the GRN inference pipeline. CellOracle (Kamimoto *et al.* 2023) constructs a cis-regulatory network from scATAC-seq data and refines the network using scRNA-seq data through a linear regression model. scMTNI (Zhang *et al.* 2023) is a probabilistic graphical model that infers cell-type-specific GRNs using cell lineage tree, scATAC-seq data, and scRNA-seq data. scMultiomeGRN (Xu *et al.* 2025), on the other hand, uses graph neural networks to fuse the regulatory knowledge provided by different modalities and infers GRNs for the cell population. Most of these methods, regardless of various algorithm details, follow the same two-step inference backbone: they first use scATAC-seq data to construct a “prior graph” that incorporates the possible regulatory events between TFs and target genes, and then refine the “prior graph” using scRNA-seq data. However, since they still focus on population-level GRN inference, the “prior graph” is still constructed through population-level region information within scATAC-seq data, which fails to consider the variations of open chromatin regions between cells. On the other hand, spatial transcriptomics (ST) data provides the spatial location of cells along with the gene expression data. Functionally similar cells tend to form spatially layered structures within many tissues (Ma *et al.* 2022), indicating that spatially close cells can have similar GRNs in these tissues.

In this paper, we propose CeSpGRN (Cell-Specific Gene Regulatory Network inference), a computational method that infers cell-specific GRNs from single-cell multi-omics and spatial data. Typical GRN inference methods require a sufficient number of cells to infer one GRN, which makes inferring cell-specific GRNs for all cells seemingly impossible. In CeSpGRN, we assume that the GRNs of cells change smoothly along the cell trajectory or spatial landscape. This assumption enables “information borrowing” across different cells: when inferring the GRN for a cell, CeSpGRN uses data from not only the cell itself, but also its neighboring cells. The neighboring cells in CeSpGRN are defined according to the similarity of gene expression data or spatial location. With the “information borrowing” strategy, CeSpGRN does not require additional trajectory inference or cell clustering steps that were used in many high-resolution GRN inference methods (Wang *et al.* 2023a, Zhang *et al.* 2023), and circumvents the potential error introduced by these steps. CeSpGRN uses a Gaussian Copula Graphical Model (GCGM) (Liu *et al.* 2012, Wang *et al.* 2014) to model the gene expression data. GCGM is expanded upon the Gaussian Graphical Model (GGM). Compared to GGM, GCGM accounts for the non-Gaussian nature of single-cell gene expression data. The design of CeSpGRN makes it possible to apply the method to single-cell multi-omics datasets. Given the paired scATAC-seq and scRNA-seq data where both modalities are simultaneously measured for each cell, CeSpGRN learns the cell-specific prior GRNs from the scATAC-seq data and refines the cell-level prior GRNs with the scRNA-seq data. When scATAC-seq data is not available, CeSpGRN constructs prior GRNs from transcription factor information instead. Given ST data, CeSpGRN can also incorporate the spatial information into the weighted kernel construction.

We tested CeSpGRN on three real datasets covering different GRN inference scenarios, including a paired scATAC-seq and scRNA-seq dataset, a scRNA-seq dataset, and an ST dataset. We also quantitatively measure the performance of CeSpGRN on simulated datasets. The test result shows the broad applicability of CeSpGRN and its superior performance in reconstructing dynamically rewired regulations.

## 2 Materials and methods

### 2.1 Overview of CeSpGRN

**Figure 1:**
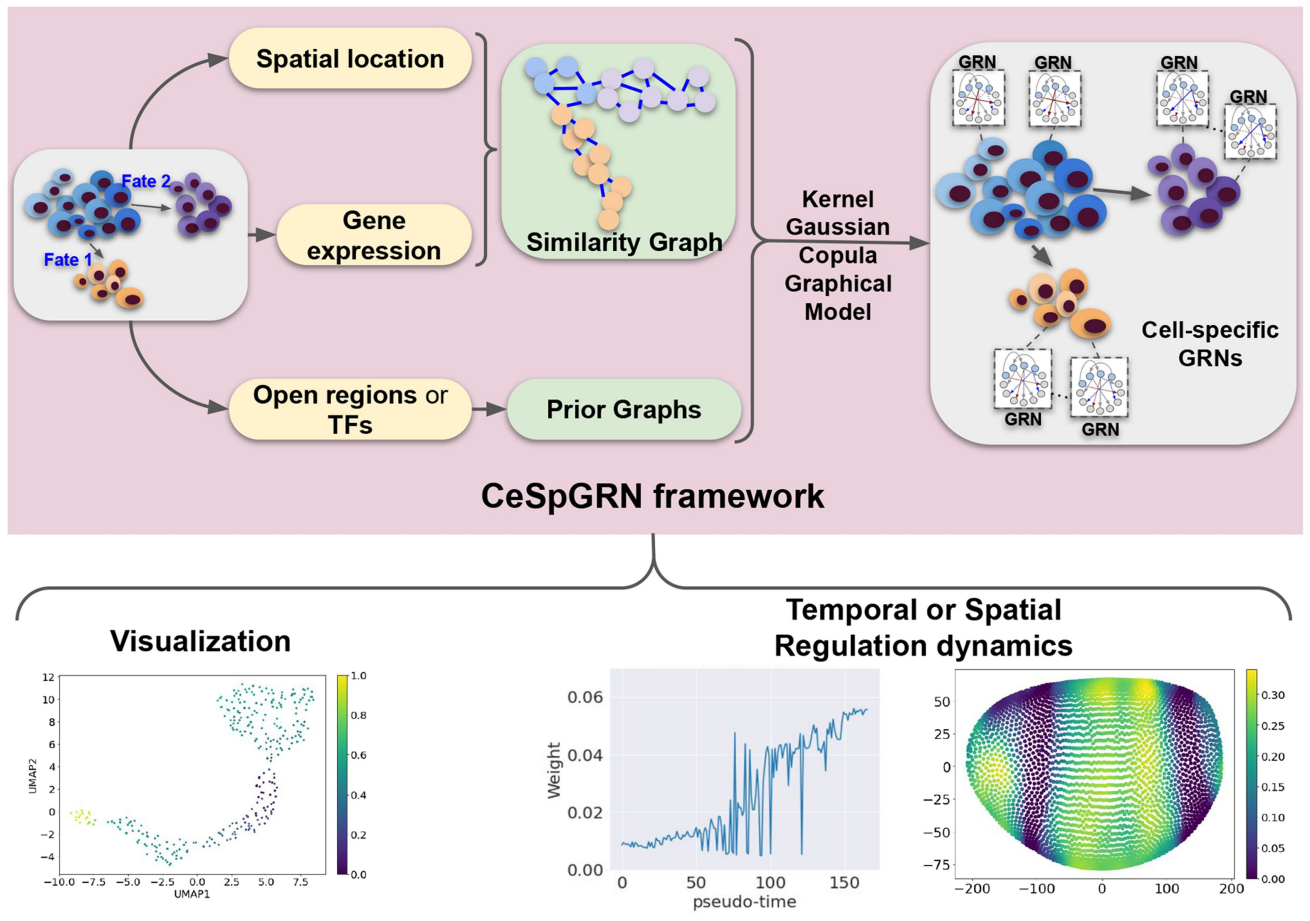
Overview of CeSpGRN framework. CeSpGRN can be applied to paired scATAC-seq and scRNA-seq data, or ST data. It infers cell-specific GRNs, which can be used as a cell feature for visualization of cell latent space, as well as to study regulatory dynamics across time or space.

CeSpGRN works in two steps: (i) construction of kernel weights and (ii) inference of cell-specific GRNs using kernel weights (Fig. 1). CeSpGRN is built upon the basic assumption that the cells’ GRNs change smoothly along the cell trajectory, and the cells that are close to each other should have similar GRNs (termed “smooth-changing” assumption). CeSpGRN first constructs a *k*-nearest neighborhood (*k*-NN) graph using gene expression data. Notably, the “smooth-changing” assumption does not need to be on the gene expression space: it can be further extended to encode the spatial adjacency information, in cases where GRNs are considered to change smoothly along the spatial landscape. The *k*-NN graph is constructed using the spatial location of cells. The kernel weights are then calculated by applying a Gaussian kernel function on the *k*-NN graph (Section 2.2). The kernel weight between cells *i* and *j* is denoted as $K_{ij}$.

We then construct the objective function of CeSpGRN using the kernel weights. For cell *i*, given the gene expression data $X_{i}$ and the corresponding GRN $\Theta_{i}$ (belonging to the positive definite set $S_{++}^{g}$), the likelihood function can be written as $\ell_{\Theta_{i}}(X_{i})$. According to the “smooth-changing” assumption, the expression value of cell *j* from the neighborhood of cell *i* ($j\in N_{i}$) should also partially reflect the GRN $\Theta_{i}$. We denote the likelihood of observing $X_{j}$ given $\Theta_{i}$ as $\ell_{\Theta_{i}}(X_{j})$. The objective function of cell *i* is a weighted average of the likelihood function of itself and its neighboring cells:


(1) $$\begin{array}{c} {\hat{\Theta }}_{i}=\arg\underset{\Theta_{i}\in S_{++}^{g}}{\min}-\sum_{j\in N_{i}}K_{ij}\ell_{\Theta_{i}}(X_{j}) \end{array}.$$

If cell *j* lies further to cell *i*, then the gene expression of cell *j* should reflect less of $\Theta_{i}$. This is controlled by a smaller kernel weight $K_{ij}$ that is applied on $\ell_{\Theta_{i}}(X_{j})$ in the weighted average. Similarly, cell *j* lies closer to cell *i*, it should reflect more of $\Theta_{i}$, which means a larger kernel weight. The likelihood function $\ell_{\Theta_{i}}(\cdot )$ is calculated using GCGM (Section 2.4). We further incorporate the $\ell_{1}$ regularization on $\Theta_{i}$ to control the sparsity of the inferred graph. When the chromatin accessibility data or TF information are available for the cells, cell-specific prior GRN can be constructed and incorporated into the objective function (Section 2.3). The prior GRN significantly reduces the search space of GRN, and we design an additional regularization term using the prior GRN to eliminate the impossible connections of GRN. Denoting the binary adjacency matrix of prior GRN in cell *i* as $G_{i}^{\mathrm{prior}}$, we can calculate the mask matrix of cell *i* by conducting an element-wise reverse: $M_{i}=1-G_{i}^{\mathrm{prior}}$. The final objective function is


(2) $$\begin{array}{c} {\hat{\Theta }}_{i}=\arg\underset{\Theta_{i}\in S_{++}^{g}}{\min}-\sum_{j\in N_{i}}K_{ij}\ell_{\Theta_{i}}(X_{j})+\lambda \|\Theta_{i}{\|}_{1}+\beta \|M_{i}⊙\Theta_{i}{\|}_{F}^{2} \end{array}.$$

$\lambda \|\Theta_{i}{\|}_{1}$ is the $\ell_{1}$ sparsity regularization on $\Theta_{i}$ and λ is the hyperparameter. $M_{i}⊙\Theta_{i}$ denotes the element-wise multiplication of $M_{i}$ and $\Theta_{i}$, and the term $\beta \|M_{i}⊙\Theta_{i}{\|}_{F}^{2}$ penalizes the connections that are not included in the prior GRNs. β is the regularization weight that controls the strength of this constraint.

The objective function is solved separately for each cell *i* in the dataset using ADMM algorithm (Section 2.4). The values in the inferred $\Theta_{i}$ cannot be readily used as the edge weights of the GRN. We further transform $\Theta_{i}$ into a partial correlation matrix $G_{i}$ (Section 2.5), where a larger absolute partial correlation value at the (m,n)th element of $G_{i}$ means a stronger regulation interaction between genes *m* and *n*.

Major hyperparameters of CeSpGRN include the kernel bandwidth σ, neighborhood size $N_{i}$, sparsity regularization weight λ, and prior information regularization weight β. Hyperparameter selection is discussed in Note 1.4, available as supplementary data at *Bioinformatics* online.

### 2.2 Constructing kernel-weighted matrix

The kernel weight $K_{ij}$ is constructed to reflect the transcriptome similarity between cell *i* and cell *j*. We adapt the idea from manifold learning (Moon *et al.* 2019, Zhang *et al.* 2022) to calculate $K_{ij}$ for every pair of cells. We first perform principal component analysis (PCA) on the scRNA-seq data matrix, and calculate pairwise Euclidean distance between cells using their low-dimensional PCA representations. We then construct a *k*-NN graph between cells using the pairwise distance matrix. We are then able to approximate the manifold distance between every two cells by calculating the corresponding geodesic distance (Tenenbaum *et al.* 2000) between them on the *k*-NN graph. Denote the geodesic distance between cells *i* and *j* by $D_{ij}$, the kernel weight $K_{ij}$ is then calculated using a Gaussian kernel function:


(3) $$K_{ij}=\text{exp}(-D_{ij}^{2}/(2\sigma^{2})),$$

where σ is the bandwidth hyperparameter that accounts for the differences between cell GRNs. A larger σ means that GRN is changing more slowly between cells, and a smaller σ means that GRN is changing faster. The selection of the number of neighbors in the *k*-NN graph, *k*, is discussed in Note 1.4, available as supplementary data at *Bioinformatics* online.

In the case where the “smooth-changing” assumption is valid on the spatial landscape of cells in ST data, $D_{ij}$ is the Euclidean distance between the spatial locations of cells, and the rest of the procedure to calculate $K_{ij}$ remains the same.

### 2.3 Constructing prior GRNs using chromatin accessibility data or TF knowledge

When scATAC-seq data of each cell is provided, we can construct prior GRNs for each cell using the chromatin accessibility information. Given an accessible chromatin region, we can connect the region to the target gene using the relative location between regions and genes on the genome. In our test, we consider the regions that lie within 50 kb upstream of the target gene TSS to be the regions that connect to the target gene. On the other hand, the region can be connected to the transcription factor using the motif information in the region. According to the accessible regions, we can connect the transcription factors to the corresponding target genes. We term the constructed graph between transcription factors and target genes to be the prior GRN. Different sets of regions are accessible in different cells. As a result, the constructed prior GRN is also cell-specific (Fig. 1, available as supplementary data at *Bioinformatics* online). We construct the binary adjacency matrix of the prior GRN in cell *i* as $G_{i}^{\text{prior}}$, where 0 element means the interaction does not exist at the corresponding entry and 1 otherwise.

When the paired scATAC-seq data is not provided, CeSpGRN can still construct a prior GRN using the TF information, i.e. which genes are TFs and which genes are target genes. When transforming the TF information into the prior GRN $G_{i}^{\mathrm{prior}}$, we set all entries corresponding to the interaction between target genes to be 0, and all remaining entries to be 1 (TF and TF, TF and Target). That is because the interactions in GRN are mainly between TF and TF, or TF and the target gene.

### 2.4 Inferring cell-specific GRNs with weighted GCGM

In this section, we introduce the GCGM and the detailed objective function of CeSpGRN (Equation 2). GCGM expands upon the GGM. GGM assumes that the data follows a multivariate Gaussian distribution, and the underlying graph is encoded in the precision matrix of the model. However, GGM is limited to data with Gaussian distribution. GCGM is then developed to better account for the non-Gaussian nature of the data. In GRN inference, we assume the gene expression data is observed from GCGM, and we aim to infer the precision matrix that corresponds to the GRN. Inferring the precision matrix of a GGM or GCGM from the observed data has been studied by previous work (Dobra *et al.* 2004, Friedman *et al.* 2008, Banerjee *et al.* 2008, Liu *et al.* 2012, Wang *et al.* 2014).

Denoting the gene expression data of cell *i* as a vector $X_{i}\in R^{g}$, where *g* is the number of genes, and the precision matrix of the undirected GRN as Θ. The log-likelihood function of GCGM can be written as:


(4) $$\begin{array}{c} \ell_{\Theta }({\left\{X_{i}\right\}}_{i=1}^{n})=-\frac{n}{2}\text{log}\;\det(\Theta )+\frac{n}{2}tr(\hat{\Sigma }\Theta ) \end{array}.$$

*n* is the number of cells and $\hat{\Sigma }$ is the *nonparametric covariance matrix* of GCGM. We then include the log-likelihood function of GCGM (Equation 4) into the objective function of CeSpGRN (Equation 2):


(5) $$\begin{array}{cc} {\hat{\Theta }}_{i}=\arg\underset{\Theta_{i}\in S_{++}^{g}}{\min}\frac{\left|N_{i}\right|}{2}\sum_{j\in N_{i}}K_{ij}\left[-\text{log}\;\det(\Theta_{i})+tr({\hat{\Sigma }}_{j}\Theta_{i})\right] & \\ & +\lambda \|\Theta_{i}{\|}_{1}+\beta \|M_{i}⊙\Theta_{i}{\|}_{F}^{2} \end{array}.$$

The remaining problem is the estimation of *nonparametric covariance matrix* $\hat{\Sigma }$. In the original GCGM model, $\hat{\Sigma }$ is calculated based on Kendall’s τ correlation (Liu *et al.* 2012, Wang *et al.* 2014). In CeSpGRN, we need to calculate ${\hat{\Sigma }}_{j}$ for every cell *j*. We again borrow information from neighboring cells of cell *j*, and estimate ${\hat{\Sigma }}_{j}$ (Wang *et al.* 2014) using a neighborhood-weighted Kendall’s τ correlation score:


(6) $$\begin{array}{cc} & \tau_{j}(m,n)=\frac{\sum_{\overset{k,k^{\prime }\in N_{j}}{k\neq k^{\prime }}}w_{kk^{\prime }}^{mn}\text{sign}((X_{k}^{m}-X_{k^{\prime }}^{m})(X_{k}^{n}-X_{k^{\prime }}^{n}))}{\left|N_{j}|\cdot (|N_{j}|-1\right)}\\ & {\hat{\Sigma }}_{j}(m,n)=\{\begin{array}{cc} \text{sin}\;\frac{\pi }{2}\tau_{j}(m,n) & \text{if}\;m\neq n\\ 1 & \text{if}\;m=n \end{array} \end{array},$$

where $w_{kk^{\prime }}^{mn}=b_{k}^{m}b_{k^{\prime }}^{m}b_{k}^{n}b_{k^{\prime }}^{n}K_{kk^{\prime }}$. $K_{kk^{\prime }}$ is the kernel weight between cells *k* and $k^{\prime }$ (following Equation 3). $b_{k}^{m},b_{k^{\prime }}^{m},b_{k}^{n},b_{k^{\prime }}^{n}$ are binary annotation for the zero values: $b_{k}^{m}=1$ if the expression of gene *m* in cell *k*, $X_{k}^{m}$, is not zero, and $b_{k}^{m}=0$ if $X_{k}^{m}=0$. The estimated ${\hat{\Sigma }}_{j}$ is not guaranteed to be positive definite, and we project ${\hat{\Sigma }}_{j}$ into the positive definite sets by replacing its non-positive eigenvalues with a small positive value $\epsilon =10^{-3}$. $N_{j}$ is the set of neighboring cells set of cell *j*. The size of neighborhood $\left|N_{j}\right|$ is a hyperparameter in the formula, with a similar role as that of the bandwidth parameter σ.

Having calculated kernel weight $K_{ij}$ and ${\hat{\Sigma }}_{j}$, we now can proceed to solve the optimization problem (Eq. 5). We use the ADMM algorithm to perform the optimization, as (i) ADMM preserves the positive definiteness of $\Theta_{i}$ for each iteration as long as the initial $\Theta_{i}$ is positive definite (proof in Note 1.2, available as supplementary data at *Bioinformatics* online); (ii) ADMM quickly converges to a reasonably good suboptimum (Boyd *et al.* 2011); (iii) The convergence condition and hyperparameter choices for the algorithm are well-studied (Boyd *et al.* 2011, Nishihara *et al.* 2015). To apply ADMM, we transform the original optimization problem into the following problem:


(7) $$\begin{array}{c} {\hat{\Theta }}_{i},Z=\arg\underset{\Theta_{i},Z\in S_{++}^{g}}{\min}\frac{\left|N_{i}\right|}{2}\sum_{j\in N_{i}}K_{ij}\left[-\text{log}\;\det(\Theta_{i})+tr({\hat{\Sigma }}_{j}\Theta_{i})\right]\\ +\lambda \|Z{\|}_{1}+\beta \|M_{i}⊙Z{\|}_{F}^{2}\\ s.t.\;\Theta_{i}=Z \end{array}.$$

The new optimization problem in Equation 7 can be solved with Algorithm 1 (pseudo-code in Note 1.1, available as supplementary data at *Bioinformatics* online).

### 2.5 Calculating the partial correlation matrix

The zero entries in the inferred precision matrix $\Theta_{i}$ means conditional independence of the corresponding genes. However, the non-zero values of $\Theta_{i}$ cannot be directly used to quantify the regulation strength of genes. We introduce an additional step to transform the precision matrix $\Theta_{i}$ into the partial correlation matrix $G_{i}$. Given the (m,n)th entry of $\Theta_{i}$, the corresponding $G_{i}(m,n)$ can be calculated as


(8) $$G_{i}(m,n)=-\frac{\Theta_{i}(m,n)}{\sqrt{\Theta_{i}(m,m)\Theta_{i}(n,n)}}.$$

The values of the partial correlation matrix reveal the regulation strength between genes, where a larger value means the corresponding genes have a stronger regulation relationship.

## 3 Results

### 3.1 Benchmark on simulated datasets

We first benchmark the accuracy of CeSpGRN using simulated datasets, and compare it with baseline GRN inference methods. We consider methods under two different GRN inference scenarios, where (1) scRNA-seq data and TF information are available and (2) scRNA-seq data and scATAC-seq data are available. CeSpGRN is applicable for both inference scenarios. Under Scenario 1, we compare CeSpGRN with scMTNI (Zhang *et al.* 2023), GENIE3 (Huynh-Thu *et al.* 2010), SCODE (Matsumoto *et al.* 2017), CSN (Dai *et al.* 2019), and LocCSN (Wang *et al.* 2021). Under Scenario 2, we compare CeSpGRN with SCENIC+ (Bravo González-Blas *et al.* 2023), scMTNI (Zhang *et al.* 2023), CellOracle (Kamimoto *et al.* 2023), and scmultiomeGRN (Xu *et al.* 2025). scMTNI infers cluster-level GRNs using scATAC-seq and scRNA-seq data. When scATAC-seq is not available, scMTNI infers GRNs using scRNA-seq data and TF information. “TF information” refers to the information on which genes are TFs. Here we denote the scMTNI that uses TF information and scATAC-seq data as “scMTNI (TF)” and “scMTNI (ATAC)”, respectively. GENIE3 is a tree-based inference method that infers population-level GRNs from scRNA-seq data and TF information, and was among the top-performing methods that infer GRN from scRNA-seq data (Pratapa *et al.* 2020). SCODE learns the GRN from cell differentiation dynamics using both scRNA-seq data and pseudotime information. CSN and LocCSN infer a cell-specific gene association graph from scRNA-seq data through pairwise statistical tests. SCENIC+ is constructed from a tree-based GRN inference algorithm, GRNboosts2, and incorporates the prior knowledge from scATAC-seq as inference constraints. CellOracle is a linear regression model that infers cluster-level GRNs using scATAC-seq and scRNA-seq data. scmultiomeGRN is a deep learning algorithm that combines the information from scATAC-seq and scRNA-seq data using graph neural networks. We evaluate the GRN inference accuracy for each individual cell separately. For the population-level GRN inference accuracy, we use the population-level GRN as the GRN of each individual cell within the population. The running details of the baseline methods are described in the Note 1.7, available as supplementary data at *Bioinformatics* online. The simulated datasets are generated using scMultiSim (Li *et al.* 2025). The simulation procedure is described in Note 1.8, available as supplementary data at *Bioinformatics* online.

**Figure 2:**
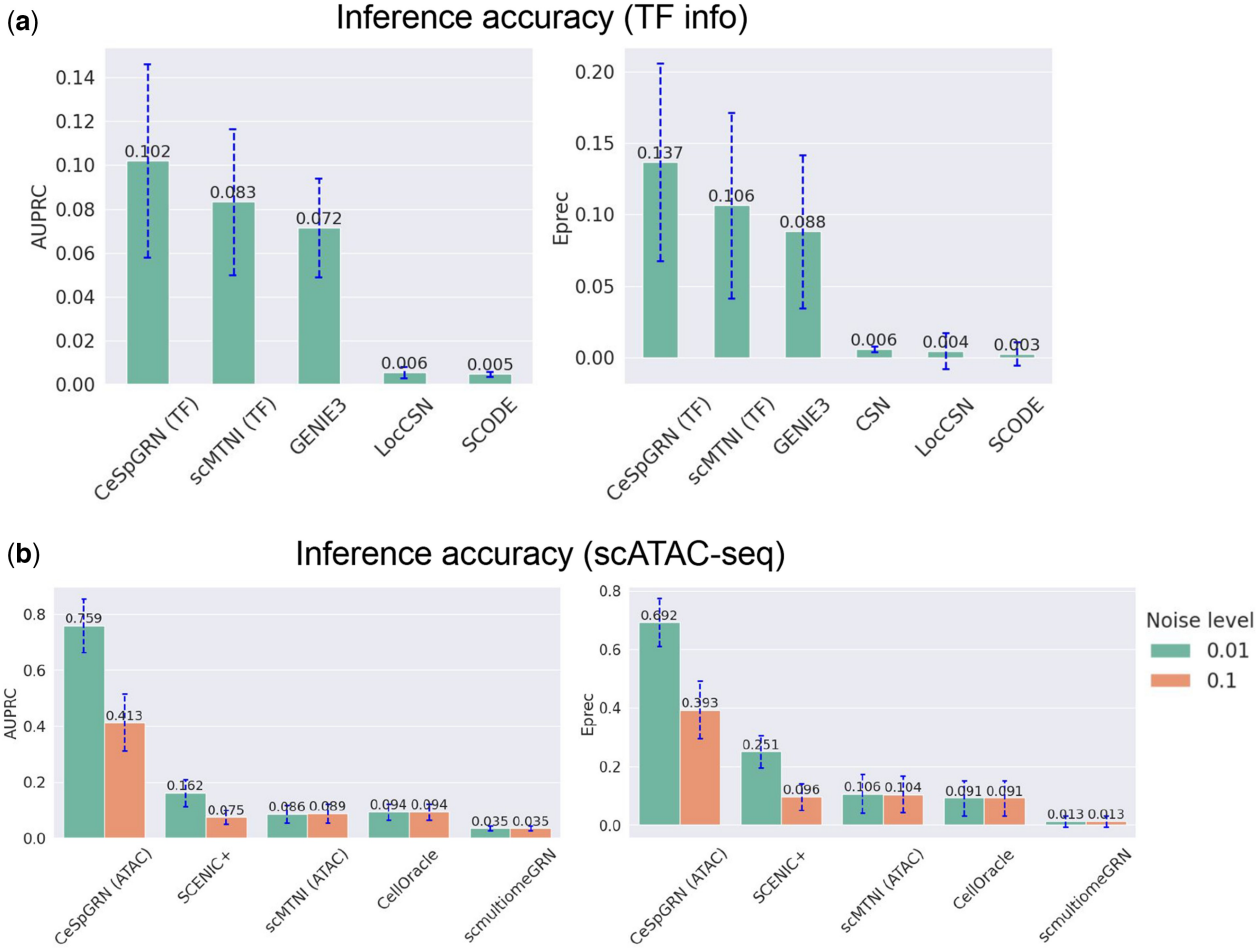
Results on simulated datasets. (a) The AUPRC and early precision (Eprec) scores of CeSpGRN and baseline methods when TF information is provided. The AUPRC score of CSN is not provided since CSN only generates binary relationships instead of continuous scores between genes. (b) The AUPRC and early precision (Eprec) scores of CeSpGRN and baseline methods when scATAC-seq data is provided. “Noise level” represents the error rate of the association between the chromatin regions and genes.

We first tested the methods under Scenario 1. We generated three scRNA-seq datasets with different random seeds, and then benchmarked the performance of CeSpGRN (denoted as “CeSpGRN (TF)”) and four baseline methods, scMTNI (TF), GENIE3 (TF), CSN and SCODE. CellOracle is not included here because it requires scATAC-seq data. We measured the inference accuracy using the Area Under the Precision-Recall Curve (AUPRC) score and the early precision scores (Note 1.8, available as supplementary data at *Bioinformatics* online). In the result (Fig. 2a), we observed that CeSpGRN (TF) has higher AUPRC and early precision scores, which shows that CeSpGRN (TF) better recovers the dynamically changed GRNs from scRNA-seq data. CSN and SCODE perform the worst compared to the other methods since they do not use the TF information in their inference framework.

We further tested the methods under Scenario 2. We generated six paired scRNA-seq and scATAC-seq datasets with different random seeds and two different noise levels of “cross-modality relationship” (0.01 and 0.1, term defined in Note 1.8, available as supplementary data at *Bioinformatics* online). The result (Fig. 2b) shows that CeSpGRN (ATAC) performs clearly better compared to the baseline methods. This is because CeSpGRN (ATAC) utilizes the accessible regions in each cell for cell-specific prior network construction, whereas the other methods only use the bulk-level chromatin accessibility information. On the other hand, the noise levels of cross-modality relationship also affect the overall performance of CeSpGRN (ATAC) (Fig. 2b). This is because a higher noise level means that the prior networks constructed from scATAC-seq data are less accurate, which affects the overall performance of CeSpGRN. Overall, the benchmark results on simulated datasets demonstrated the capability of CeSpGRN in inferring GRNs at single-cell resolution.

On the other hand, it is noteworthy that CeSpGRN and baseline methods all show a significant performance gain under Scenario 2 compared to Scenario 1. The availability of scATAC-seq information boosts the overall model inference accuracy. Since the activated TF-target gene interactions are linked to the open chromatin regions in scATAC-seq data, a large portion of false positive TF-target gene interactions can be eliminated with the scATAC-seq data, thereby narrowing the search space of potential GRNs.

### 3.2 CeSpGRN infers cell-specific GRNs from paired scRNA-seq and scATAC-seq dataset

**Figure 3:**
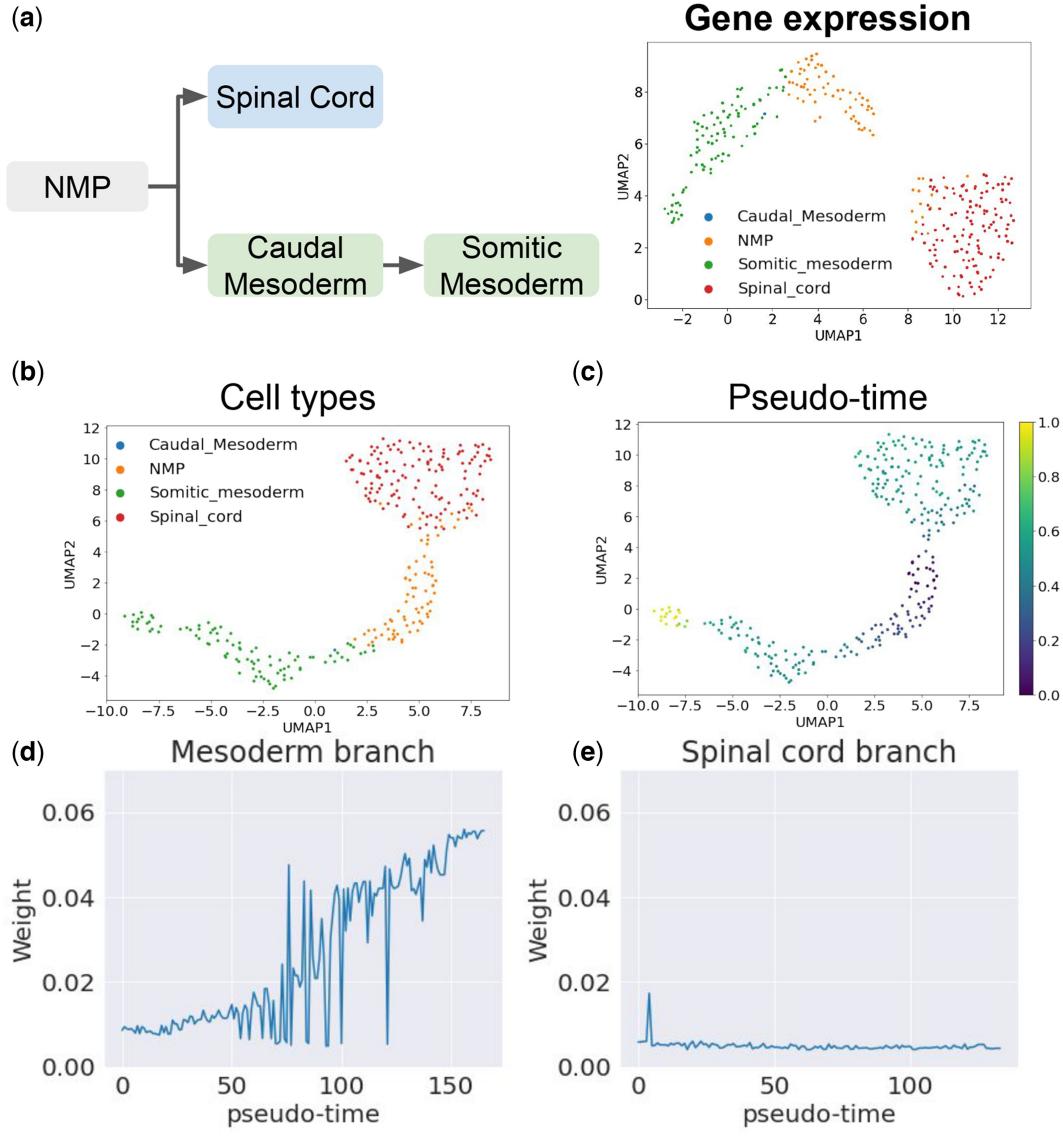
Results on NMP differentiation dataset. (a) (Left) The differentiation trajectory of NMP cells. (Right) UMAP visualization of gene expression data. (b and c) UMAP visualization of inferred GRNs, where cells are annotated by (b) the original cell-type annotation, and (c) inferred diffusion pseudotime. (d) Change of edge weight between *Mesp2* and *Ripply2* along the two differentiation fates. The regulatory strength increases along the Mesoderm branch but remains low along the Spinal cord branch.

We then applied CeSpGRN to a paired scATAC-seq and scRNA-seq dataset of mouse neuromesodermal progenitors (NMPs) differentiation (Argelaguet *et al.* 2022). The dataset measures both chromatin accessibility and gene expression data for each individual cell within NMP population. NMPs are a type of bipotent stem cell that can differentiate into elongating spinal cord cells and somitic mesoderm cells (Argelaguet *et al.* 2022). Here, we randomly sampled 300 cells from the dataset (in consideration of both obtaining noticeable differences between GRNs between single cells and running time) that cover both differentiation fates of NMPs (Fig. 3a), and selected 610 genes that include both highly variable genes and key transcription factors obtained from the motif information within the scATAC-seq dataset (Note 1.3, available as supplementary data at *Bioinformatics* online).

We constructed cell-specific prior GRNs from the scATAC-seq data (Section 2.3), and then ran CeSpGRN using the prior GRNs and gene expression data. We visualized the inferred GRN matrices using UMAP, and colored each matrix using the cell-type annotation of its corresponding cells in the original literature (Argelaguet *et al.* 2022) (Fig. 3b). From the UMAP visualization, we observed that the inferred cell-specific GRNs preserve the bifurcating trajectory of NMPs, where one branch leads to the spinal cord cells (red cells in Fig. 3b) and the other branch leads to the somite mesoderm cells (green cells in Fig. 3b). We then analyzed the highly variable hub genes under each differentiation branch, which are the genes whose regulatory interactions are highly dynamic. The highly variable hub genes are selected according to the variance of the total edge weights that connect to each gene along the branch, and a larger variance means that the corresponding gene shows more rewiring activity in the differentiation process. We selected the top 100 hub genes for each branch and ran gene ontology analysis using topGO (Alexa and Rahnenführer 2021) on each set of genes. The top gene ontology terms of each branch are shown in Table 1, available as supplementary data at *Bioinformatics* online. Both branches include the term “anterior/posterior axis specification”, which is highly relevant to NMPs differentiation (Sambasivan and Steventon 2020). In the somite mesoderm branch, we also found a highly relevant GO term “mesoderm development.”

Cells in the mesoderm trajectory are affected by the regulation between genes *Mesp2* and *Ripply2* (Morimoto *et al.* 2007). During the somitogenesis process, the expression of *Mesp2* induces the expression of *Ripply2* which in return regulates the total abundance of *Mesp2*. The regulation results in the spatial periodicity of the segmented somites (Morimoto *et al.* 2007). Here, we analyzed the change in regulation weight between *Mesp2* and *Ripply2* along both branches. We first inferred the developmental pseudotime of each cell using the diffusion pseudotime (DPT) algorithm (Haghverdi *et al.* 2016) (Fig. 3c). Then, we sorted the cells according to the inferred pseudotime along each branch and plotted the edge weight between *Mesp2* and *Ripply2*, inferred by CeSpGRN, following the sorted order of cells (Fig. 3d). Along the mesoderm branch, it can be clearly observed that the weight increases and becomes more significant in somitic mesoderm cells, whereas the weight remains 0 along the spinal cord branch. The result shows the branch-specific regulation between *Mesp2* and *Ripply2*, which further validates the inferred GRNs.

### 3.3 CeSpGRN detects regulation dynamics in mouse embryonic stem cells

We further applied CeSpGRN to a mouse embryonic stem cell (mESC) dataset (Klein *et al.* 2015). In this dataset, 2717 mESC were sequenced using scRNA-Seq. The dataset includes cells from the following developmental stages: “before leukemia inhibitory factor (LIF) withdrawal”, “2 days after LIF withdrawal”, “4 days after LIF withdrawal”, and “7 day after LIF withdrawal”. These four stages correspond to four cell types, respectively, labeled as “mES cells”, “day 2”, “day 4” and “day 7”. With the LIF withdrawal, cells start to differentiate. The dataset records the onset of differentiation where cells fluctuate between a pluripotent inner cell mass-like state and a differentiating epiblast-like state.

**Figure 4:**
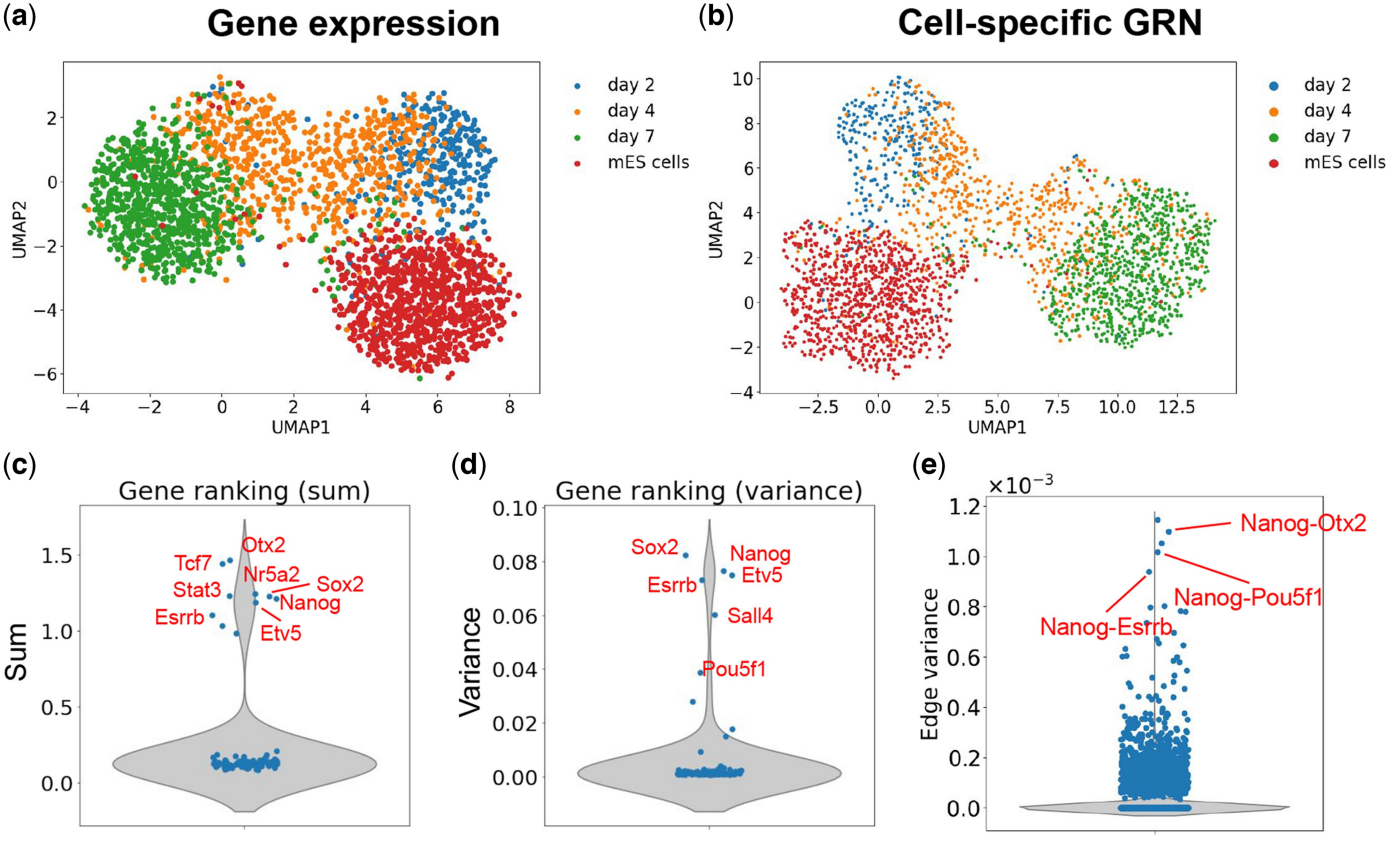
Results on mouse embryonic stem cells dataset. (a) UMAP visualization of gene expression. Cells colored by known cluster labels. (b) UMAP visualization of cell-specific GRNs inferred by CeSpGRN-TF. Dots colored by the cells’ labels. (c) Violin plot showing the total edge weight connecting to each gene. Genes with high total weights are overall highly active, and the highlighted genes are known to be key genes in mESC differentiation. (d) Violin plot showing the variance of edge weight connecting to each gene. Genes with highly variable edge weights tend to be changing their regulatory activities during differentiation. The highlighted genes are known to be involved in mESC differentiation. (e) Violin plot showing the variance of edge weights. Highlighted interactions have the most variable weights during differentiation.

We preprocessed the dataset and selected 96 key genes which are highly variable and also included in the key regulatory gene list reported by Zhou *et al.* (2007) for mESCs (Note 1.3, available as supplementary data at *Bioinformatics* online). Among the 96 genes, 10 are known to be TFs (*Pou5f1*, *Nr5a2*, *Sox2*, *Sall4*, *Otx2*, *Esrrb*, *Stat3*, *Tcf7*, *Nanog*, *Etv5*) and the remaining are target genes (Zhou *et al.* 2007). The UMAP visualization of the preprocessed gene expression data is shown in Fig. 4a. Since there is no paired scATAC-seq data but TF information, we constructed prior GRNs using the TF information and inferred cell-specific GRNs using CeSpGRN (Section 2.3). We visualized the inferred GRNs using UMAP (Fig. 4b). From the UMAP visualization, we observe a clear differentiation trajectory matching the trajectory pattern of gene expression data (Fig. 4a and b).

The cell-specific GRNs inferred by CeSpGRN allow us to analyze the dynamics of GRNs in this dataset. First, we calculate the means and variances of total edge weights connecting to each gene across all inferred GRNs. We sort the genes according to their mean and variance values separately. The mean and variance measure two different aspects of genes: a larger mean total edge weight means that the gene potentially has more regulatory activities, whereas a larger variance means that the gene rewires more often in the cell population. We observed several genes that rank at the top in both evaluating metrics, including *Otx2*, *Tcf7*, *Nanog*, etc. These genes were shown to play crucial roles in mESC development (Zhou *et al.* 2007, Acampora *et al.* 2013) (Fig. 4c and d). We also check the variance of edge weights and select the edges that vary the most in the cell population. CeSpGRN detected top-ranking edges including *Pou5f1* (*Oct4*)-*Nanog*, *Esrrb*-*Nanog*, and *Nanog*-*Otx2* (Fig. 4e). Those edges were discussed as the key interactions for mESC differentiation (Loh *et al.* 2006, Zhou *et al.* 2007, Acampora *et al.* 2017, Sevilla *et al.* 2021).

We further check how the total number of target genes of the key regulators changes along the differentiation trajectory. We infer the cell developmental pseudotime using DPT (Haghverdi *et al.* 2016). For each cell along the trajectory, we then calculate the total number of target genes connecting to the key regulators by thresholding the edge weights. We then plot the target gene number of regulators *Nanog* and *Sox2* along the trajectory (Fig. 2a and b, available as supplementary data at *Bioinformatics* online). For both genes, we observed an overall increasing trend in edge weights along the trajectory. Interestingly, we observe that for both genes, the initial target gene numbers are much smaller in “mES cells” cluster and increase gradually along the differentiation path. The trend also matches the experiments, where LIF is withdrawn after “mES cells” and cells start to differentiate.

### 3.4 CeSpGRN detects spatially associated regulation changes from ST data

CeSpGRN can also be applied to ST data to study GRN dynamics in space. We applied CeSpGRN to a Drosophila embryo dataset (Karaiskos *et al.* 2017). The dataset measures the spatial transcriptome of 84 genes from 3039 cells. We use the 3D locations of cells in the space to construct the weighted kernel function in CeSpGRN (Section 2.2). We then infer the cell-specific GRNs among the 84 genes with the gene expression measurements. Using the inferred GRN, we then study the spatial specificity of gene regulation during the Drosophila embryo morphogenesis.

**Figure 5:**
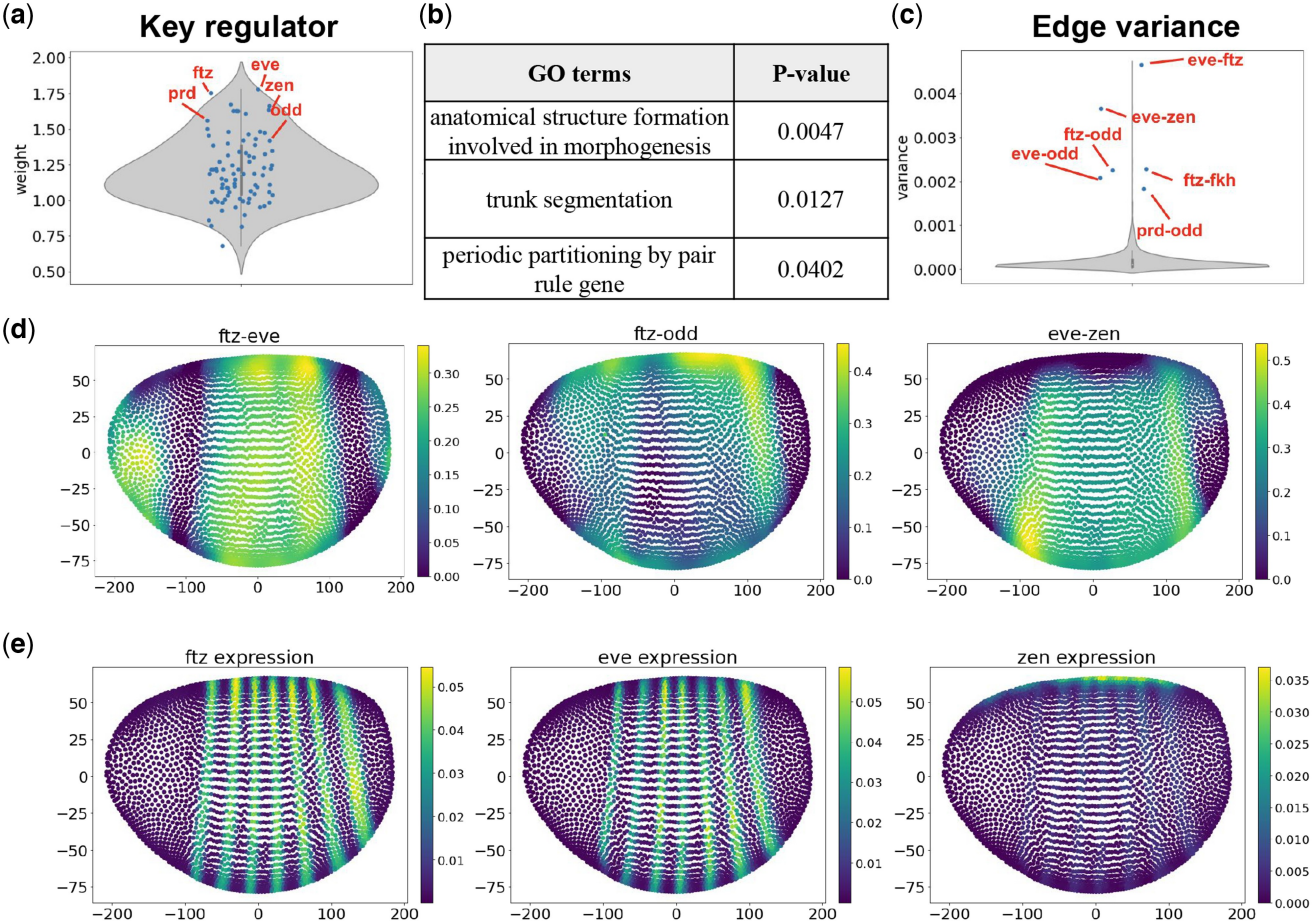
Result on Drosophila embryo spatial gene expression dataset. (a) Violin plot showing the total weights for each gene in the dataset. The marked genes have high total weights and are reported to drive the morphogenesis process. (b) The gene ontology term corresponds to the key regulators with the largest weight. Significant terms are related to embryo development and segmentation. (c). Violin plot showing the distribution of edge variance across space. Genes involved in frequently rewiring edges are known to regulate the morphogenesis process. (d). The spatial distribution of edge weights, including edges “ftz-eve”, “ftz-odd”, and “eve-zen”. These interactions are shown to be active in different spatial regions of the Drosophila embryo. (e) The spatial distribution of gene expression, including genes *ftz*, *eve*, and *zen*.

We first explore the key regulators that drive the developmental process. We calculate the total absolute edge weights that connect to each gene for each cell. We then average the total absolute edge weights across cells and rank the genes according to the average weights. Among the top-scoring genes, we found multiple transcription factors that were reported to drive the morphogenesis process, including multiple pair-rule genes *eve*, *odd*, *prd*, and *ftz* (Fig. 5a). We then selected the top 20 genes and conducted the gene ontology analysis using *topGO*. We discovered multiple terms related to embryo development and segmentation: “anatomical structure formation involved in morphogenesis”, “trunk segmentation”, etc. (Fig. 5b). We then explore the edges that rewire the most across space. We calculate the variance of edge weights for each edge in the inferred GRNs, and rank the edges according to their variances. We found highly variable edges between *ftz* and *eve*, *eve* and *zen*, *odd* and *eve*, etc. Interestingly, most of the highly variable edges includes genes *eve* and *ftz*, and these two genes were reported to bind to most genes in Drosophila embryos in order to regulate the morphogenesis process (Liang and Biggin 1998) (Fig. 5c).

We further select several highly variable edges connecting to key regulators *eve*, *odd*, and *ftz*, and visualize the weight distributions of these edges across space (Fig. 5d). Given the segmented structure of the Drosophila embryo, to capture a more detailed shift of regulation weight corresponding to the structural change, we used the edge weights inferred with a small bandwidth (0.01) instead of the GRNs aggregated across all parameters. We visualize the spatial distribution of weights corresponding to edges “ftz-eve”, “ftz-odd”, and “eve-zen” in Fig. 5d. From the visualization, we observe a strong spatial specificity of those edges across space. *ftz* and *eve* are important transcription factors related to embryonic segmentation, and the distribution of their expression level also follows the anatomical segmentation structure of Drosophila embryo (Liang and Biggin 1998, Karaiskos *et al.* 2017) (Fig. 5e). We observe a similar segmentation pattern in the weight distribution between *ftz* and *eve* (Fig. 5d, left), indicating that the regulation strength between “ftz-eve” can also follow the segmentation structure of the Drosophila embryo. Gene *zen* controls the development of dorsal-ventral pattern in Drosophila embryo, and it is mainly expressed in the dorsal part of the embryo (Doyle *et al.* 1989) (Fig. 5d, right). Interestingly, from the weighted distribution of “eve-zen” (Fig. 5d, right), we observed a complementary pattern, where the regulation weights are stronger in the ventral part and weaker in the dorsal part. While these results are hard to validate given the scarcity of knowledge on spatial trends of regulatory dynamics, these predictions can serve as initial conjectures, and further evidence can be developed with experimental tests or future computational methods.

### 3.5 Ablation test

#### 3.5.1 Ablation test on GCGM

To prove the effectiveness of GCGM, we conduct an ablation test by comparing the performance of CeSpGRN using the current weighted version of GCGM [covariance matrix follows Equation 6, termed “CeSpGRN (GCGM)”] with CeSpGRN using the original unweighted GCGM [termed “CeSpGRN (GCGM-ori)”] and using GGM [termed “CeSpGRN (GGM)”]. We test the models using the 6 simulated datasets where both scATAC-seq and scRNA-seq data are available (Scenario 2 of Section 3.1), and measure the model performance using AUPRC and early precision scores. The result of both models is shown in Fig. 3a, available as supplementary data at *Bioinformatics* online. Compared to CeSpGRN (GCGM), CeSpGRN (GCGM-ori) has consistently lower scores in both evaluation metrics. CeSpGRN (GGM) has significantly lower AUPRC scores compared to CeSpGRN (GCGM). Even though the average early precision score of CeSpGRN (GGM) is slightly higher than CeSpGRN (GCGM), its performance is more unstable with larger score deviation. The result shows that the use of GCGM significantly stabilizes the model performance and improves the overall model inference accuracy.

#### 3.5.2 Ablation test on the sparsity regularization

The $\ell_{1}$ sparsity regularization term $\lambda \|\Theta_{i}{\|}_{1}$ controls the sparsity of the inferred GRNs, where a larger regularization weight λ means that the inferred graph should be sparser. We conduct the ablation test of the sparsity regularization term using the simulated datasets under Scenario 1 of Section 3.1. We select datasets under Scenario 1 (only scRNA-seq is available) to prevent the scATAC-seq-related regularization term from affecting the ablation test result. We compare the inference accuracy of CeSpGRN with and without the $\ell_{1}$ sparsity regularization term [named “CeSpGRN” and “CeSpGRN (w/o lambda)”], and the result is shown in Fig. 3b, available as supplementary data at *Bioinformatics* online. AUPRC score is not improved by the sparsity regularization, but the early precision score is higher with the use of the sparsity regularization. The result is expected, since the early precision score measures the accuracy of the top-scoring edges, and the sparsity regularization promotes the significant edge selection of the inference algorithm.

#### 3.5.3 Ablation test on the scATAC-seq-related regularization

The scATAC-seq-related regularization $\beta \|M_{i}⊙\Theta_{i}{\|}_{F}^{2}$ controls how much the prior knowledge provided by scATAC-seq affect the final GRN inference result, where a larger regularization weight β means that the prior GRN obtained from scATAC-seq has a higher influence on the final GRN inferred by CeSpGRN. We conduct the ablation test of the scATAC-seq-related regularization term using the 6 simulated datasets from Scenario 2 of Section 3.1, and measure the model performance using AUPRC and early precision scores. The result is shown in Fig. 3c, available as supplementary data at *Bioinformatics* online, where the model “CeSpGRN” performs significantly better than the model “CeSpGRN (w/o beta).” The result shows that the use of scATAC-seq-related regularization term removes the erroneous edges inferred from scRNA-seq data and significantly improves the model inference accuracy.

### 3.6 Computational scalability and hyperparameter evaluation of CeSpGRN

We further analyze the running time scalability of CeSpGRN on datasets of different numbers of cells and genes, and evaluate the robustness of CeSpGRN with the hyperparameter selection. The analysis are included in Notes 1.5 and 1.6, available as supplementary data at *Bioinformatics* online.

## 4 Discussion and future work

We proposed CeSpGRN, a method that infers cell-specific GRNs from single-cell multi-omics or spatial data. Although inferring one GRN for every single cell can be a computationally heavy task, the assumption that closely located cells (in the gene expression space or spatial landscape) have similar GRNs can significantly reduce the parameter space of the cell-specific GRNs inference problem. CeSpGRN constructs objective functions for each cell using a weighted GCGM, where the weights are calculated based on a *k*NN graph constructed from either the gene expression profiles or the spatial location of the cells. When paired scATAC-seq data is available, CeSpGRN innovatively constructs the cell-specific prior GRNs and uses the prior GRNs to improve the accuracy of GRNs. The construction of cell-specific prior GRNs fully explores scATAC-seq data by using the region accessibility of each cell. The design of CeSpGRN allows it to be broadly applied to scRNA-seq data, paired scRNA-seq and scATAC-seq data, and ST data. We demonstrated the performance of CeSpGRN using real and simulated datasets that cover various application scenarios and showed that CeSpGRN can detect key regulators and regulations that rewire across cells.

CeSpGRN, being the first method that infers cell-specific GRNs using single-cell multi-omics data, has its limitations. First of all, the assumption that cells in a neighborhood have similar GRNs may not apply to scenarios where the change of cell states is abrupt, even between closely located cells (gene expression or spatial location). In the future, CeSpGRN may be generalized to incorporate new knowledge on the dynamic pattern of GRNs over space. In addition, CeSpGRN infers undirected graphs, whereas gene regulations are directional in reality. The directions can be easily resolved for interactions between a TF and a non-TF gene, but not for interactions between TFs. We envision that more methods that infer single-cell resolution GRNs will be proposed to resolve these limitations.

## Supplementary Material

btag324_Supplementary_Data

## Data Availability

The datasets that are analyzed within the current study are publicly available. The raw NMP differentiation dataset is available on Gene Expression Omnibus with the accession code GSE205117. The raw mESC dataset is available on Gene Expression Omnibus with the accession code GSE65525. The Drosophila embryo dataset is available on Gene Expression Omnibus with the accession code GSE95025. The source code of CeSpGRN is available on GitHub (https://github.com/PeterZZQ/CeSpGRN), and published on Zenodo (https://doi.org/10.5281/zenodo.19189926).
