# Supplementary material for: CeSpGRN: inferring cell-specific gene regulatory networks from single-cell multi-omics and spatial data
Source: Bioinformatics. 2026 Jun 1;42(6):btag324. doi: 10.1093/bioinformatics/btag324 (PMC13242928; doi:10.1093/bioinformatics/btag324)
Supplement: btag324_Supplementary_Data [file btag324_supplementary_data.pdf]

Supplementary file: CeSpGRN: Inferring cell-specific gene regulatory networks from single cell multi-omics and spatial data

1 Supplementary Notes

1.1 Pseudo-code of CeSpGRN

Algorithm 1 CeSpGRN algorithm

function CeSpGRN($\mathbf{K}$, $\{\hat{\mathbf{\Sigma}}_i\}_{i=1}^n$, $\mathbf{M}$, t_{max}) // t_{max} is the max number of iterations
  $\mathbf{\Theta} = \{\}$ // store GRN for all cells
  for i **in** $1, 2, \cdots, n$ **do**
    Initialize $\bar{\mathbf{\Sigma}}_i = \frac{1}{\rho}\sum_{j=1}^n \mathbf{K}_{ij} \cdot \hat{\mathbf{\Sigma}}_j$; $\mathbf{U}^1 = \mathbf{0}$
    Initialize $\mathbf{Z}^1 = \text{diag}(\bar{\mathbf{\Sigma}}_i)$ // ensure symmetric positive definite
    for t **in** $1, 2, \cdots, t_{max}$ **do**
      $\mathbf{\Theta}_i^{t+1} = \sqrt{\frac{1}{\rho}\mathbf{I} + \frac{1}{4}(\mathbf{U}^t - \mathbf{Z}^t + \bar{\mathbf{\Sigma}}_i)^T(\mathbf{U}^t - \mathbf{Z}^t + \bar{\mathbf{\Sigma}}_i)} - \frac{1}{2}(\mathbf{U}^t - \mathbf{Z}^t + \bar{\mathbf{\Sigma}}_i)$
      $\mathbf{Z}^{t+1} = sign(\mathbf{\Theta}_i^{t+1} + \mathbf{U}^t)\left(\frac{\rho|\mathbf{\Theta}_i^{t+1}+\mathbf{U}^t|-\lambda}{\rho+2\beta\mathbf{M}^2}\right)_+$
      $\mathbf{U}^{t+1} = \mathbf{U}^t + \mathbf{\Theta}_i^{t+1} - \mathbf{Z}^{t+1}$
    $\mathbf{\Theta} = \mathbf{\Theta} \cup \mathbf{Z}^{t+1}$
  return $\mathbf{\Theta}$

Parameter ρ in the algorithm affects the convergence speed of the algorithm, and is set to be 1.7 [Nishihara et al., 2015].

1.2 Algorithms 1 preserve the positive definiteness of the inferred matrix

When we initialize $\mathbf{Z}^0$ to be symmetric positive definite, then according to algorithm 1, we will always have $\mathbf{Z}^t$ to be symmetric. Then $\frac{1}{2}(\mathbf{U} - \mathbf{Z}^t + \bar{\mathbf{\Sigma}}_i)$ is also symmetric, and can be eigenvalue decomposed

as $\mathbf{V}\boldsymbol{\Lambda}\mathbf{V}^T$, where $\mathbf{V}^T\mathbf{V} = \mathbf{V}\mathbf{V}^T = \mathbf{I}$. Rewriting the updating rule of $\boldsymbol{\Theta}_i^{t+1}$ in Algorithm 1 as

$$\begin{aligned}\boldsymbol{\Theta}_i^{t+1} &= \sqrt{\frac{1}{\rho}\mathbf{I} + \frac{1}{4}(\mathbf{U}^t - \mathbf{Z}^t + \bar{\boldsymbol{\Sigma}}_i)^T(\mathbf{U}^t - \mathbf{Z}^t + \bar{\boldsymbol{\Sigma}}_i)} \\ &- \frac{1}{2}(\mathbf{U}^t - \mathbf{Z}^t + \bar{\boldsymbol{\Sigma}}_i) \\ &= \sqrt{\frac{1}{\rho}\mathbf{I} + \mathbf{V}\boldsymbol{\Lambda}^2\mathbf{V}^T} - \mathbf{V}\boldsymbol{\Lambda}\mathbf{V}^T \\ &= \mathbf{V}\left(\left(\boldsymbol{\Lambda}^2 + \frac{1}{\rho}\mathbf{I}\right)^{\frac{1}{2}} - \boldsymbol{\Lambda}\right)\mathbf{V}^T\end{aligned} \tag{1}$$

Since $\left(\left(\boldsymbol{\Lambda}^2 + \frac{1}{\rho}\mathbf{I}\right)^{\frac{1}{2}} - \boldsymbol{\Lambda}\right)$ has minimum eigenvalue greater than 0, $\boldsymbol{\Theta}_i^{t+1}$ is always positive definite. Algorithm 1 is able to preserve the symmetric positive definiteness of $\boldsymbol{\Theta}_i^{t+1}$.

1.3 Preprocessing steps of the real datasets

Preprocessing step of NMP differentiation dataset: We constructed the cell-specific $\{G_i^{prior}\}$ directly from the raw scATAC-seq dataset following the steps described above: we connected the regions with the transcription factor (TF) motifs using the R package *Signac*, and connected the regions with the target genes using the $50kb$ distance threshold on the genome as described above. We preprocessed scRNA-seq dataset using Python package *scanpy* by first filtering out genes with a total count smaller than 30 and then selecting the top-500 highly-variable genes. We finally used the overlapping genes between the preprocessed scRNA-seq dataset and $\{G_i^{prior}\}$. We randomly select 300 cells within the dataset for GRN inference.

Preprocessing steps of mESC dataset: We picked the 4 scRNA-seq samples from the raw dataset covering several developing time points ("GSM1599494_ES_d0_main.csv", "GSM 1599497_ES_d2_LIFminus.csv", "GSM1599498_ES_d4_LIFminus .csv", and "GSM1599499_ES_d7_LIFminus.csv"). We select the top-4000 highly variable genes within the scRNA-seq dataset using scanpy, and further pick the overlapping genes between the dataset and the key regulatory gene list reported by Zhou et. al [Zhou et al., 2007]. The final gene set, including 96 genes, among which 10 genes are known to be the TFs.

We log-normalized the gene expression data in all real datasets before applying CeSpGRN.

1.4 Hyper-parameter selection

The number of neighbors in the k-NN graph, k, is set to be the minimum number larger than 5 that makes the graph connected. We select this k value because a k value that is too large makes the estimated manifold distance vulnerable to short-circuit errors, and a k value that is too small tends to fragment the data manifold into disconnected regions [Balasubramanian et al., 2002]

Other hyper-parameters in CeSpGRN include the kernel bandwidth σ, neighborhood size N_i, sparsity regularization weight λ, and prior information regularization weight β. The kernel bandwidth σ and neighborhood size N_i control the change speed of cell GRNs, where a smaller σ and N_i mean GRNs change faster across the cell population. λ controls how sparse the inferred GRN is, whereas β controls how close the inferred GRNs are to the prior GRNs. In our test, we fix $\beta = 1$ and $N_i = 100$. We scan through $\sigma = [0.01, 0.1, 1, 10]$ and $\lambda = [0.01, 0.05, 0.1, 0.5]$ and infer GRNs under each parameter setting. The final GRN is obtained by averaging the inferred GRNs under each parameter setting.

1.5 Evaluation on the scalability of CeSpGRN

We test how the running time of CeSpGRN scale with the number of genes and cells in scRNA-seq dataset. Firstly, we set the number of genes to 110, and measure the running time of CeSpGRN on datasets with number of cells equal to 160, 800, 2000, 4000, 8000, and 16000, and compare it with baseline methods including LocCSN, CellOracle, GENIE3, SCENIC+, scmultiomeGRN, scMTNI, and SCODE. Since different methods infers different number of GRNs from the cell population, we compare the methods with regard to both their total inference time and their averaged inference time per GRN. The running time curve of CeSpGRN and baseline methods follows Fig. S5. Then, we set the number of cells to 400, and measure the total and per-GRN running time of CeSpGRN and baseline methods on datasets with number of genes equal to 27, 55, 110, 550, and 770. The running time curve of CeSpGRN and baseline methods follows Fig. S6. The running time of CeSpGRN increases with both the number of genes and the number of cells. Although CeSpGRN exhibits a relatively longer total runtime compared with the baseline methods, this is because CeSpGRN infers one GRN for each cell in the dataset. When considering the runtime per inferred GRN, CeSpGRN demonstrates competitive computational efficiency compared with the baseline methods.

1.6 Hyper-parameter test of CeSpGRN

The hyper-parameters in CeSpGRN includes: (1) kernel bandwidth σ, (2) Neighborhood size $|N_i|$, (3) sparsity regularization weight λ, and (4) scATAC-seq-related regularization weight β. To account for the hyper-parameter selection of λ and σ, the inferred GRNs of CeSpGRN is the ensemble of GRNs under parameter combinations: $\sigma = \{0.01, 0.1, 1, 10\}$ and $\lambda = \{0.01, 0.05, 0.1, 0.5\}$. However, the effect of β and $|N_i|$ on the model performance still remain to be examined. We randomly select one simulated dataset from the simulated datasets generated in section 3.1, and run CeSpGRN with $\beta = \{0.01, 0.1, 1, 10, 100\}$ and $N = \{5, 15, 30, 50\}$. We evaluated the model performance under different hyper-parameter settings using AUPRC and Early Precision scores.

Fig. S4a shows the inference accuracy of CeSpGRN with β of different values. The AUPRC and Early Precision scores do not vary significantly with the selection of β, which shows the robustness of CeSpGRN with regard to the selection of β.

Fig. S4b shows the model performance with $|N_i|$ of different values. The AUPRC and Early Precision scores of CeSpGRN with $|N_i| = 10$ is significantly lower than other $|N_i|$s, this is because the sample size is too small. $|N_i| \geq 100$ shows comparable performance, where $|N_i| = 100$ shows the best performance.

1.7 Running details of baseline methods

When running scMTNI, we first separate the cell population into 3 distinct clusters according to the simulation pseudotime. Then we ran scMTNI following the same pipeline in its online tutorial (`https://github.com/Roy-lab/scMTNI`). We set the argument "-q" to 5 when running the method with scATAC-seq data, which increases the influence of prior network on the final result. We adjust the "branch-specific gain/loss rate" in the cell lineage tree to 0.001 according to the ground truth GRN changing rate in the simulated datasets.

When running SCENIC+, we extract the TF by region and region by target gene matrices directly from scMultiSim, and insert the matrices into the SCENIC+ GRN inference function. We used the default hyper-parameter setting in the SCENIC+ algorithm following its online tutorial (`https://scenicplus.readthedocs.io/en/latest/human_cerebellum.html`).

We construct the input of scmultiomeGRN following the instruction in its code repository (`https://doi.org/10.5281/zenodo.14848389`), we construct the scATAC-seq node features using the scMultiSim generated region by target gene matrix, and construct the scRNA-seq node

features using GENIE3 inferred gene by gene matrix, and construct the prior GRN graph from scMultiSim TF by region and region by target gene matrices.

When running CellOracle, we follow the same pipeline in its online tutorial (`https://morris-lab.github.io/CellOracle.documentation/index.html`). Same as scMTNI, we also separate the cell population into 3 clusters according to the simulation pseudotime.

We run CSN and LocCSN following their online tutorials (`https://github.com/wys8c764/CSN`; `https://xuranw.github.io/locCSN/docs/vignettes.html`) with default hyper-parameter settings.

1.8 Simulation setting and evaluation metrics

We generated simulated datasets using scMultiSim. Totally 3 scRNA-seq datasets (3 random seeds: 0, 1, 2) and 6 paired scRNA-seq and scATAC-seq datasets (3 random seeds: 0, 1, 2, and 2 noise levels: 0.01, 0.1) are generated. Each dataset has a total of 8000 cells and 110 genes. For paired scRNA-seq and scATAC-seq data, the noise level measures the amount of false positive connections in the cross-modality relationship matrices (which includes two matrices: the region by TF matrix that represents the regions each TF can bind to, and the region by target gene matrix that represents which regions are associated with each target gene). A higher noise level means that the relationship matrix is less accurate, which also affect the accuracy of prior GRNs since they are constructed from the cross-modality relationship matrix.

We evaluate the GRN inference accuracy of different methods using AUPRC score and Early Precision score. Both metrics were used in the previous benchmarking paper [Pratapa et al., 2020] to measure the closeness of the inferred GRNs towards the ground truth GRNs, where a higher score means that the inferred result is more accurate. Since CeSpGRN infers cell-specific GRNs, we measure the inference accuracy of each cell separately and average the scores across all cells. We also conducted the same cell-specific evaluation for the baseline methods to make the benchmarking result comparable across methods.

2 Supplementary Figures and Tables

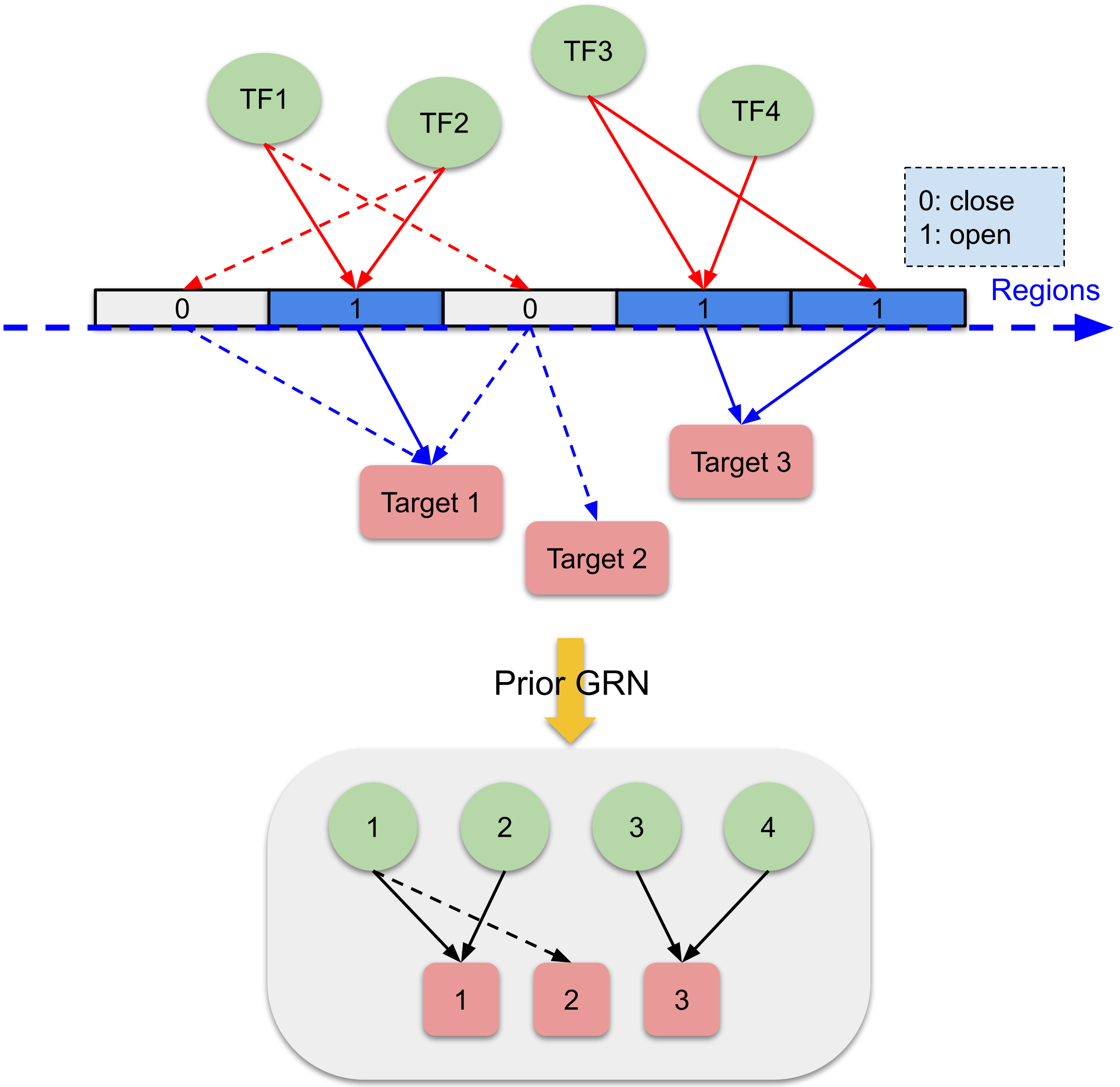

Figure S1: Graphical illustration of constructing prior GRN from chromatin accessibility data within a cell. Connections between TFs and targets are only preserved when the connecting regions are accessible. Solid lines: connections are preserved. Dashed lines: connections are removed since the regions are closed.

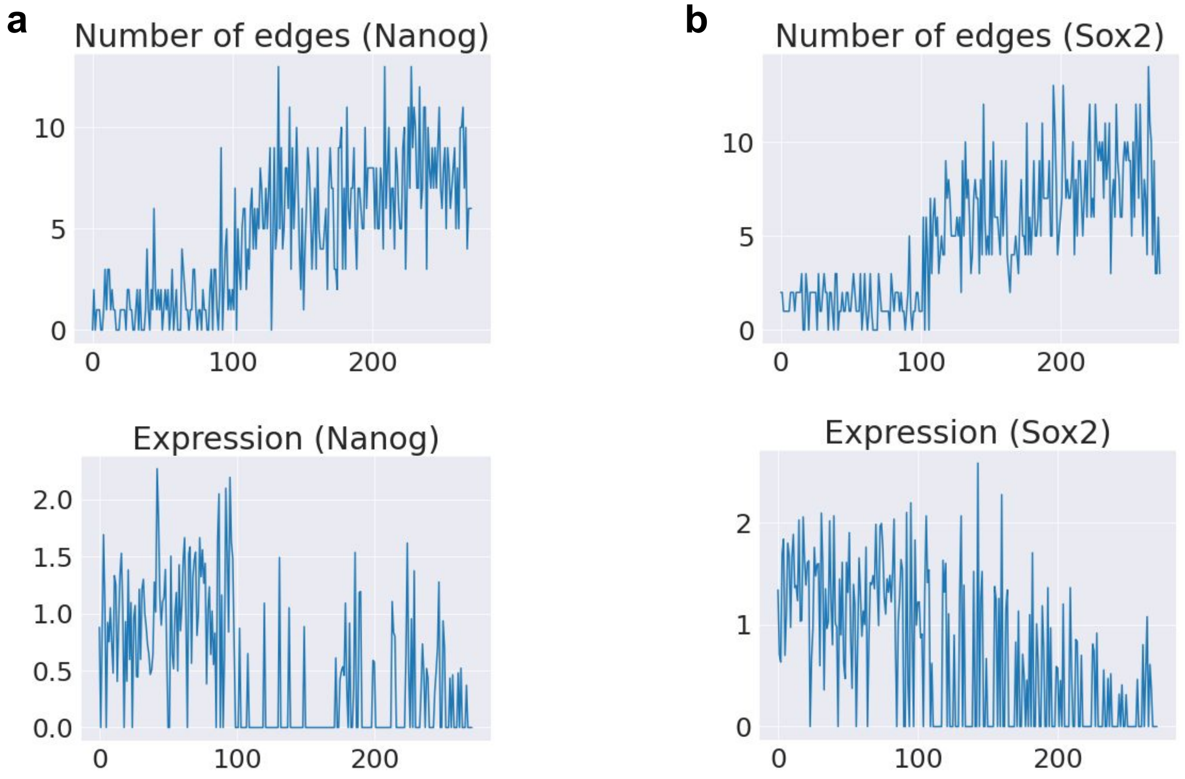

Figure S2: Results on mouse embryonic stem cells dataset. (a) The change of (upper) total edge number connecting to *Nanog* and (lower) *Nanog* expression level along the trajectory. The total number of connections has an overall increasing trend along the trajectory, as LIF is withdrawn, though the gene expression level does not increase. (b) The change of (upper) total edge number connecting to *Sox2* and (lower) *Sox2* expression level along the trajectory. The total number of connections has an overall increasing trend along the trajectory, as LIF is withdrawn, though the gene expression level does not increase. These results show that studying cell-specific GRNs show regulatory dynamics that can not be observed by analysis at the gene expression level.

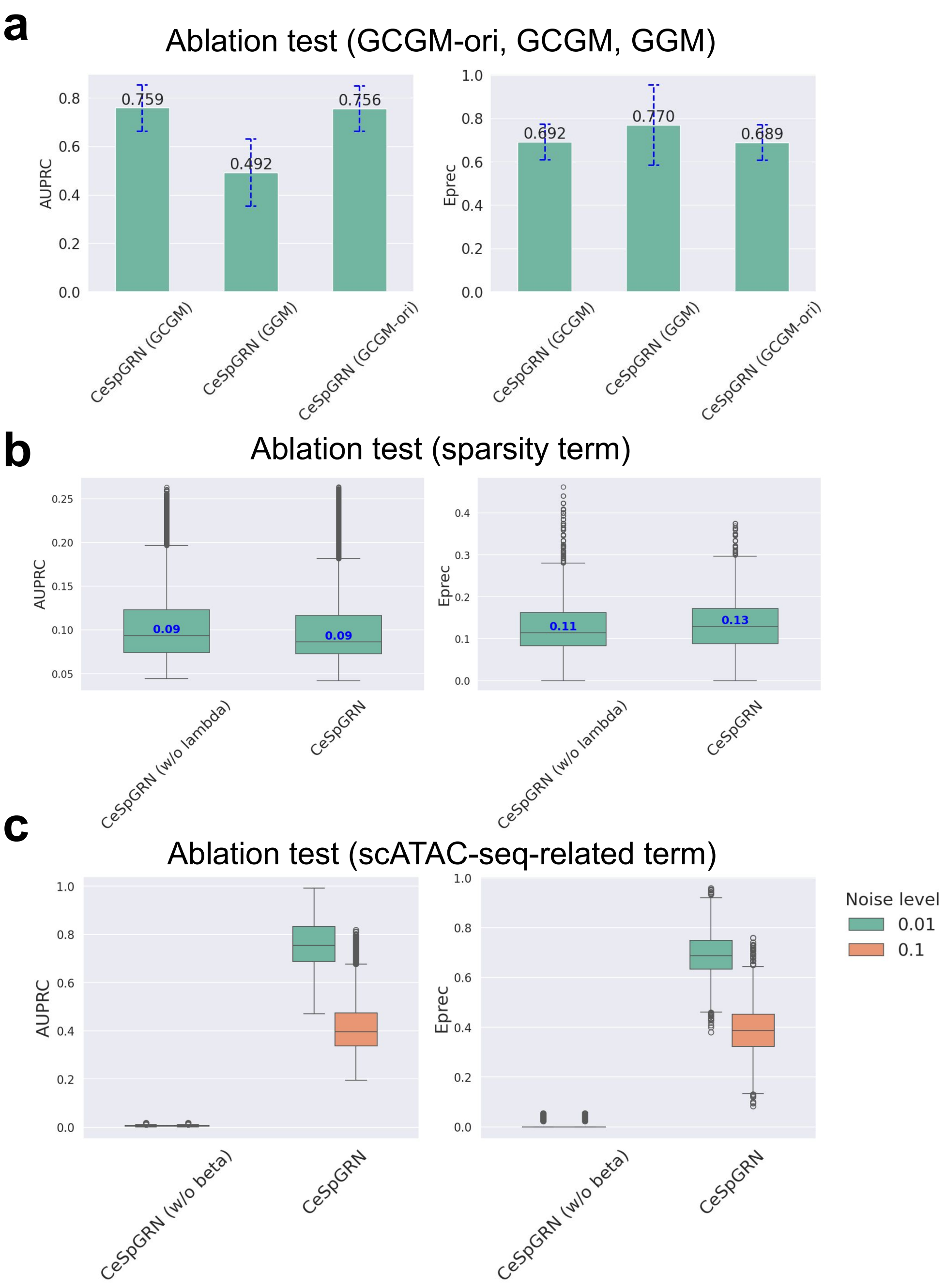

Figure S3: Ablation test results. (a) The AUPRC and Early Precision scores of CeSpGRNs using current GCGM ("CeSpGRN (GCGM)"), the original version of GCGM ("CeSpGRN (GCGM-ori)"), and GGM ("CeSpGRN (GGM)"). (b) The AUPRC and Early Precision scores of CeSpGRN with the λ regulation term ("CeSpGRN") and without the λ regulation term ("CeSpGRN (w/o) lambda"). (c) The AUPRC and Early Precision scores of CeSpGRN with the β regulation term ("CeSpGRN") and without the β regulation term ("CeSpGRN (w/o) beta")

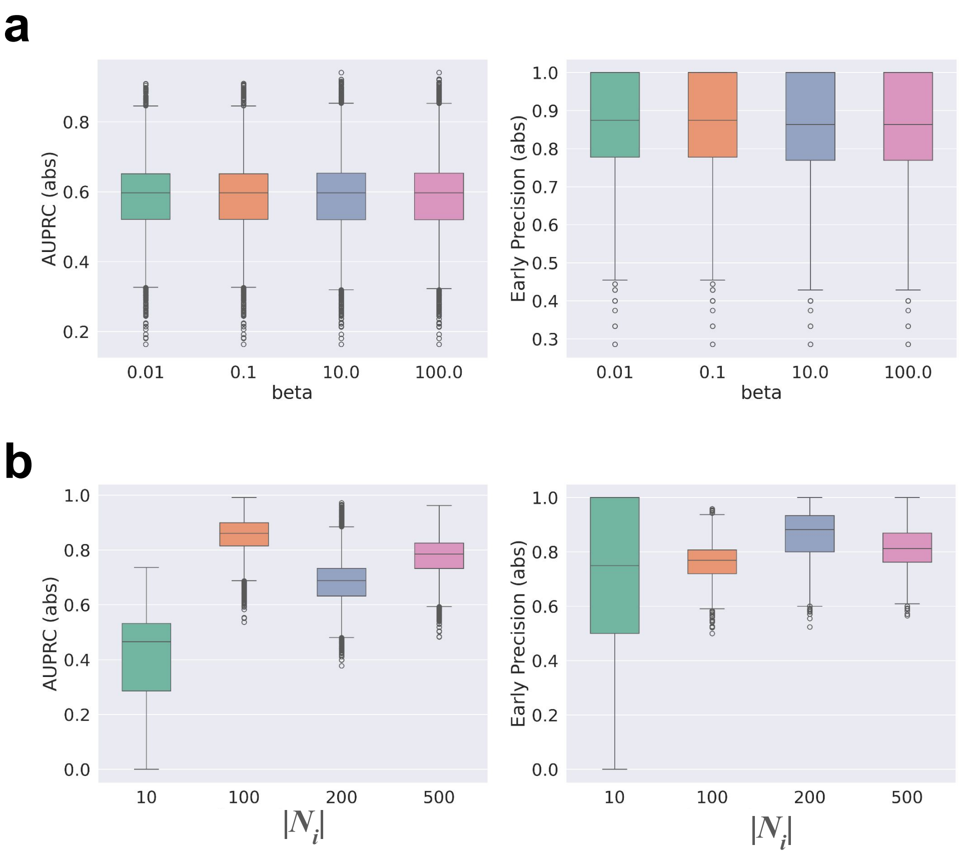

Figure S4: The hyper-parameter test results. (a) The AUPRC and Early Precision scores of CeSpGRN under different βs. (b) The AUPRC and Early Precision scores of CeSpGRN under different $|N_i|$s.

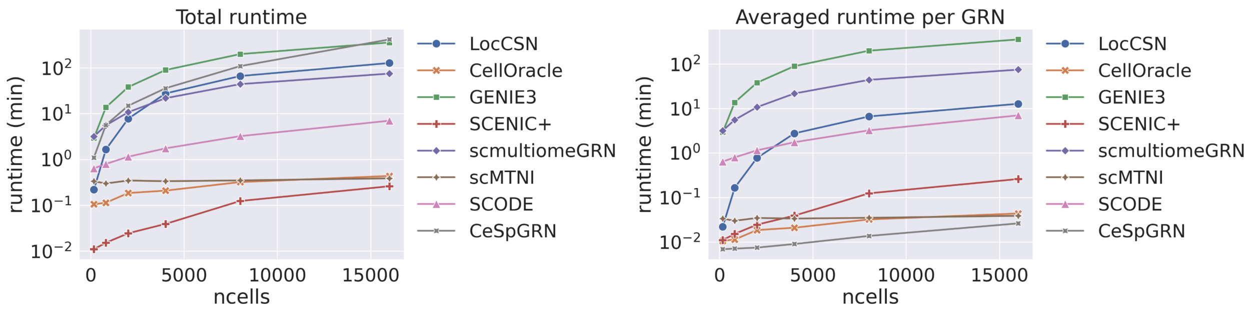

Figure S5: The inference running time of CeSpGRN and baseline methods on data with different number of cells.

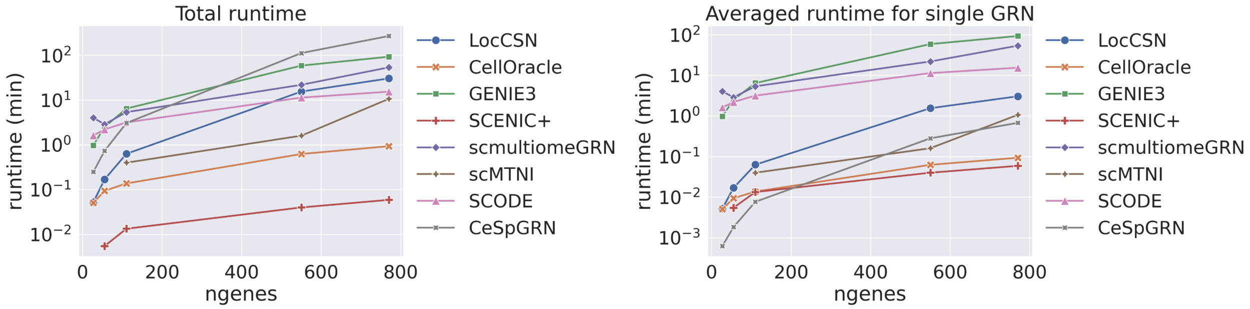

Figure S6: The inference running time of CeSpGRN and baseline methods on data with different number of genes.

GO terms	P-value	Branch
anterior/posterior axis specification	6.5e-5	Mesoderm
embryonic axis specification	0.0269	Mesoderm
mesoderm development	0.0488	Mesoderm
anterior/posterior axis specification	0.011	Spinal Cord
embryonic axis specification	0.024	Spinal Cord

Table S1: The top gene ontology terms and the corresponding p-values of two branches (Spinal Cord branch and Mesoderm branch).

References

M. Balasubramanian, E. L. Schwartz, J. B. Tenenbaum, V. de Silva, and J. C. Langford. The isomap algorithm and topological stability. *Science*, 295(5552):7–7, 2002.

R. Nishihara, L. Lessard, B. Recht, A. Packard, and M. Jordan. A general analysis of the convergence of ADMM. In *International Conference on Machine Learning*, pages 343–352. PMLR, 2015.

A. Pratapa, A. P. Jalihal, J. N. Law, A. Bharadwaj, and T. Murali. Benchmarking algorithms for gene regulatory network inference from single-cell transcriptomic data. *Nature methods*, 17(2): 147–154, 2020.

Q. Zhou, H. Chipperfield, D. A. Melton, and W. H. Wong. A gene regulatory network in mouse embryonic stem cells. *Proceedings of the National Academy of Sciences*, 104(42):16438–16443, 2007.
